# High doses of tigecycline are associated with satisfactory plasmatic and pulmonary concentrations for the treatment of severe infections due to fully susceptible bacteria: do we need even higher doses in patients under CRRT?

**DOI:** 10.1186/s13613-020-00758-5

**Published:** 2020-10-14

**Authors:** Patrick M. Honore, Leonel Barreto Gutierrez, Luc Kugener, Sebastien Redant, Rachid Attou, Andrea Gallerani, David De Bels

**Affiliations:** ICU Dept, Centre Hospitalier Universitaire Brugmann-Brugmann University Hospital, Place Van Gehuchtenplein, 4, 1020 Brussels, Belgium

We read with great interest the article by De Pascale et al. who show that the use of high doses (200 mg loading dose followed by 100 mg two times a day) of tigecycline (TGC) is associated with satisfactory plasmatic and pulmonary concentrations for the treatment of severe infections due to fully susceptible bacteria [1]. One-third of the patients in the study had acute kidney injury (AKI) requiring continuous renal replacement therapy (CRRT), yet the authors claim that losses of tigecycline via CRRT were negligible, based entirely upon the work of Broeker et al. [2]. We would like to make some comments. First, the CRRT modality chosen by Broeker et al. [2] may have influenced TGC elimination. TGC clearance during continuous veno-venous hemodiafiltration (CVVHDF) was more efficient (2.71 L/h) as compared to continuous veno-venous hemodialysis (CVVHD, 1.69 L/h) [2]. Second, Broeker et al. attribute the increased clearance of TGC with CVVHDF to low plasma protein binding (recently reported as 50–70%, compared to the previously reported 11–29%), allowing better elimination through ultrafiltration [2]. This increased ultrafiltration yields a saturation coefficient of 0.79 for CVVHD and 0.90 for CVVHDF and probably higher for continuous veno-venous hemofiltration (CVVH) [2]. The removal by CVVHDF and CVVHD yield together a value of 11.2% [2]. If we look at CVVHD alone (1.69 L/h), this represents only 9% of the total body clearance (18.3 L/h) [2]. Looking at CVVHDF (2.71 L/h), this represents almost 15%. Third, TGC protein binding is affected by divalent cations such as calcium, and accordingly, regional citrate anticoagulation (RCA) might affect membrane transfer [2]. This would be suspected if convection was used in the study as it is more protein binding dependent. RCA was only used in CVVHD and not in CVVHDF, where unfractionated heparin (UHF) was used [2]. Fourth, TGC can be adsorbed by plastic labware [3] and there is a great suspicion that TGC could be adsorbed by highly adsorptive membranes (HAM) [4]. Broeker et al. used a polysulfone membrane which is poorly adsorptive; nevertheless, in their study, they observed a time delay in the effluent concentrations in one patient that may have been caused by adsorption losses inside the membrane [2]. They concluded that since the delay indicated a saturable binding, adsorption losses did not impact the dialysis clearance significantly [2]. We respectfully disagree, as most of the adsorption of small molecules does not occur at the surface of the membrane, but rather occurs inside the membrane fibers and therefore it takes more time to become saturated [5]. Indeed, Tian et al. clearly demonstrated that the absence of saturation could exclude surface adsorption, as repeated doses of amikacin resulted in further bulk adsorption [5]. Comparing the pharmacokinetics/pharmacodynamics (PK/PD) between the Broeker and the De Pascale studies, the PK/PD was much more optimal in the Broeker study, again suggesting that even higher doses may be needed in CRRT patients. It would be interesting to know which CRRT modality, type of membrane and anticoagulation were used in the De Pascale study, in order to further elucidate the effect of CRRT on the PK/PD. Overall, the conclusion that no dose adjustment is necessary during CRRT seems somewhat premature. At this time, we cannot rule out the possibility that using a higher dose such as 100 mg three times a day in patients receiving CRRT may further improve PK/PD and perhaps related mortality.

## Data Availability

Not applicable.

## Abbreviations

TGC: Tigecycline
CRRT: Continuous renal replacement therapies
CVVVHDF: Continuous veno-venous hemodiafiltration
CVVHD: Continuous veno-venous hemodialysis
CVVH: Continuous veno-venous hemofiltration
RCA: Regional citrate anticoagulation
UFH: Unfractionated heparin
PK/PD: Pharmacokinetics/pharmacodynamics
